# Flooding Tolerance of Rice: Regulatory Pathways and Adaptive Mechanisms

**DOI:** 10.3390/plants13091178

**Published:** 2024-04-23

**Authors:** Jing Wang, Mingzhen Han, Yongxiang Huang, Junliang Zhao, Chuanguang Liu, Yamei Ma

**Affiliations:** 1 College of Coastal Agricultural Sciences, Guangdong Ocean University, Zhanjiang 524088, China; wjing1129@foxmail.com (J.W.); gdouhrb@126.com (Y.H.); 2 Rice Research Institute, Guangdong Academy of Agricultural Sciences, Guangzhou 510640, China; hmz109927@163.com (M.H.); zhao_junliang@gdaas.cn (J.Z.); guyliu@126.com (C.L.); 3 Key Laboratory of Genetics and Breeding of High Quality Rice in Southern China (Co-Construction by Ministry and Province), Ministry of Agriculture and Rural Affairs, Guangzhou 510640, China; 4 Guangdong Key Laboratory of New Technology in Rice Breeding, Guangzhou 510640, China; 5 Guangdong Rice Engineering Laboratory, Guangzhou 510640, China; 6 College of Agriculture and Biology, Zhongkai University of Agriculture and Engineering, Guangzhou 510225, China

**Keywords:** *Oryza sativa* L., flooding, *CIPK15*, *SUB1A*, *SK1/SK2*, phytohormones

## Abstract

Rice is a major food crop for more than half of the world’s population, while its production is seriously threatened by flooding, a common environmental stress worldwide. Flooding leads to oxygen deficiency, which is a major problem for submerged plants. Over the past three decades, significant progress has been made in understanding rice adaptation and molecular regulatory mechanisms in response to flooding. At the seed germination and seedling establishment stages, the CIPK15-SnRK1A-MYBS1 signaling cascade plays a central role in determining rice submergence tolerance. However, from seedlings to mature plants for harvesting, *SUB1A*- and *SK1/SK2*-regulated pathways represent two principal and opposite regulatory mechanisms in rice. In addition, phytohormones, especially gibberellins, induce adaptive responses to flooding throughout the rice growth period. This review summarizes the significant adaptive traits observed in flooded rice varieties and updates the molecular genetics and mechanisms of submergence tolerance in rice.

## 1. Introduction

Flooding is one of the most destructive environmental stresses worldwide [1]. Rice, the primary staple food crop for more than half of the world’s population, is usually cultivated in paddy fields but is also affected by flooding in rain-fed and irrigated lowlands where plants face a high risk of submergence during their life cycle. Flooding can lead to two different stresses: (1) submergence stress, where the plant organ is completely under water, and (2) water-logging stress, where the plant leaf and stem are partially submerged under water [2]. Flooding is considered a compound stress, and its primary effect is restricted oxygen (O_2_) and carbon dioxide (CO_2_) availability in plant tissues, owing to the slow diffusion of gases in water [3,4]. The decline in molecular O_2_ results in the restriction of ATP synthesis and carbohydrate resources, which has major consequences for growth and survival [5].

Rice is the only crop that can successfully germinate and grow under submergence. When germinated underwater, the coleoptile elongates rapidly, whereas the growth of the roots and leaves are blocked. The rapid elongation of the hollow coleoptile acts as a snorkel, extending to the water surface, thereby obtaining more O_2_ to support seedling establishment. Thus, anaerobic germination (AG) and coleoptile elongation are two major adaptive traits during the early growth stage of rice upon submergence [6,7,8,9]. From seedlings to mature plants for harvesting, submergence seriously compromises the overall growth, development, and yield potential of rice [10]. Based on flooding regimes, rice can be roughly divided into two major ecotypes: rain-fed lowland rice that experiences shallow submergence, and deepwater rice that experiences medium to deep submergence during the monsoon season [11,12,13,14,15]. In these cases, two completely different strategies, quiescence and escape, are employed for optimal growth and survival. For example, certain landraces of lowland rice (e.g., FR13A) show flooding tolerance for up to two weeks by entering into an energy conservation mode, in which growth is temporarily arrested and energy sources are conserved for regrowth after the stress conditions subside. In contrast, deepwater rice exhibits the potential for rapid growth (especially internode elongation), consuming carbohydrate resources, so that the upper leaves rise above the water level to escape submergence [2,12,16].

From the middle of 1990 to now, the genetic basis of submergence tolerance in rice has been gradually discovered, and several major regulators such as *Submergence-1* (*SUB1*) and *SNORKEL1/2* (*SK1/2*) have been cloned and functionally characterized [17,18,19]. Recently, several studies have revealed the molecular regulatory mechanisms underlying rice submergence tolerance during the seed germination and seedling establishment stages. In this review, we highlight the significant adaptive traits observed in flooded rice varieties and update the molecular genetics and the mechanism of submergence tolerance in rice.

## 2. Tolerance of Rice to Submergence at Seed Germination and Seedling Establishment Stages and Underlying Molecular Regulatory Mechanisms

### 2.1. Metabolism and Growth Change in Response to Submergence

In traditional rice cultivation, seeds are usually germinated in the nursery, and then, well-established seedlings are transplanted into puddled fields for further growth. Recently, direct seeding, whereby rice seeds are sown directly into the soil, has become an increasingly popular cultivation method, owing to its low cost and convenience [20]. However, flooding is common under direct seeding, particularly in rain-fed areas and fields with uneven surfaces and heavy post-sowing rainfall. Flooding stress leads to unfavorable metabolic stress for plants owing to insufficient oxygen supply. Oxygen deprivation compromises the mitochondrial electron transport chain (mETC), leading to an oxidative burst and the release of sequestered calcium into the cytosol. This surge in oxidative stress activates the mitogen-activated protein kinase (MAPK) pathway, which in turn triggers multiple signaling mechanisms to circumvent the energy crisis [21,22]. Although rice exhibits a certain degree of tolerance to oxygen deficiency for AG and anaerobic seedling development (ASD), the AG/ASD capabilities of most rice varieties are poor.

**Figure 1:**
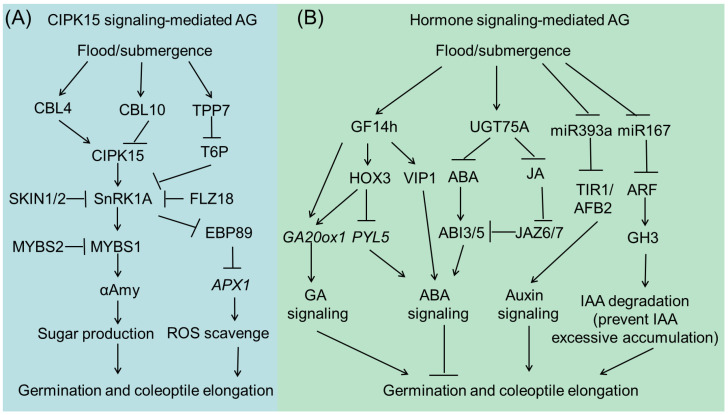
CIPK15-mediated (**A**) and phytohormone-mediated (**B**) signaling pathways in regulating rice submergence tolerance during seed germination and seedling establishment stages.

Numerous studies show that rice mainly employs ‘metabolic adaptation’ and ‘escape’ strategies to ensure seed germination and seedling development under flooded anaerobic conditions [6,9,16]. Upon submergence, starch reserves within the endosperm are hydrolyzed to produce sugars for fermentation-mediated energy production to support seed germination and seedling growth. During this process, alpha amylase enzymes, principally the AMY1 and AMY2 subfamilies, enable the degradation of starch in rice grains [23]. In air, the rice coleoptile is initially white and turns green after one day. Along with starch degradation in the endosperm, energy supply enables primary root development [24]. When germinated underwater, coleoptiles can grow for several days and rapidly elongate to reach the water surface, allowing O_2_ to diffuse into the endosperm to hydrolyze nutrients and support vigorous seedling establishment [25]. However, flooded anaerobic conditions completely inhibit root growth, which is only reinitiated as coleoptiles approach the water surface. Several important regulators and signaling cascades of rice AG/ASD have been established. CIPK15-mediated energy signaling and phytohormone signaling, including gibberellins (GA), abscisic acid (ABA), and auxin, play essential roles in determining rice seed germination and seedling establishment during submergence (Figure 1).

### 2.2. CIPK15-Mediated Sugar-Sensing Signaling in AG/AGD Regulation

It is well established that the CIPK15-SnRK1A-MYBS1 signaling cascade plays a critical role in O_2_ deficiency tolerance during rice seed germination and seedling development stages [26,27]. Submergence can trigger Ca^2+^ flux, which in turn induces conformational changes in calcineurin B-like proteins (CBLs) to allow for their hydrophobic interactions with other proteins [28]. Previous studies have demonstrated that several CBL calcium sensors (CBL2-6) can interact with CBL protein-interacting protein kinases 14 and 15 (CIPK14 and CIPK15) [29]. Recently, Ye and his co-workers found that variations in the *OsCBL10* promoter sequences contribute to this divergence in rice flooding tolerance. Flooding-tolerant rice cultivars containing the *OsCBL10* T-type promoter (which exhibits a much lower response to flooding stress than the I-type promoter) have shown lower Ca^2+^ flow and increased CIPK15 protein accumulation, *aAmy3* expression, and total aAmy activity in comparison to those in flooding-intolerant cultivars [30]. The interaction between CBLs and CIPKs activates sucrose non-fermenting 1 (Snf1)-related protein kinase 1 (SnRK1A), a global energy and stress sensor in rice, which results in the transcriptional activation and phosphorylation of MYB SUCROSE 1 (MYBS1) transcription factor. Subsequently, the expression of the starvation-induced *α*-amylase gene, *αAmy3/RAmy3D*, is activated by MYBS1 [27]. MYB SUCROSE 2 (MYBS2) competes with MYBS1 to bind and suppress the aAmy promoter during submergence [31].

Several regulators/substrates of SnRK1A have been reported to modulate rice submergence tolerance. Two hypoxia-inducible SnRK1A-INTERACTING NEGATIVE REGU-LATORs (SKIN1/2) can inhibit the expression of *MYBS1* and *aAmy3*, leading to the suppression of starch-to-sugar hydrolysis, thus delaying germination and seedling growth under submergence [32]. TREHALOSE-6-PHOSPHATE PHOSPHATASE 7 (TPP7) is another important regulator of SnRK1A, which promotes AG/ARD [33]. *TPP7* is the genetic determinant of a major quantitative trait locus (QTL), *qAG-9-2*, which catalyzes the conversion of T6P to trehalose. Reduced T6P levels release the inhibition of the CIPK15-SnRK1A-MYBS1 pathway and allow for increased starch mobilization in the form of readily fermentable sugars, thus enhancing coleoptile elongation and embryo germination [33]. Recently, a member of the FLC-like zinc finger (FLZ) protein family, FLZ18, was found to interact with SnRK1A and repress its transcriptional activation to *αAmy3*. Consistent with this, *FLZ18*-overexpressing rice plants are more sensitive to submergence than wild-type Nipponbare plants during the seed germination stage [34]. EBP89, a member of the AP2/ERF subfamily, was recently identified as a substrate of SnRK1A that can repress the expression of genes involved in the ROS-scavenging system, for example *ascorbate peroxidase 1* (*APX1*), thus negatively regulating AG/ASD [35].

### 2.3. Hormone Signaling in AG/AGD Regulation

Plant hormones are central regulators of plant growth, development, and responses to changing environments. Recent studies have revealed the critical roles of GA, ABA, and auxin in rice AG/ASD regulation. GA is a well-known hormone that can promote seed germination and seedling growth, whereas ABA represses these two processes. The balance between GA and ABA signaling determines the final seed germination performance. Recently, the 14-3-3 protein, GF14h, was shown to confer tolerance to rice AG/AGD. GF14h can interact with HOX3 and enhance its transcriptional repression activity toward downstream *PYL5*, an ABA receptor gene, to attenuate ABA signaling. GF14h can also interact with OsVP1, a transcription activator of ABA signaling, thus precisely controlling ABA response under submergence conditions. Interestingly, GF14h also promotes GA biosynthesis, possibly through the upregulation of *GA20ox1* expression, along with other unknown transcription factors. Therefore, GF14h can act as a signal switch to balance ABA signaling and GA biosynthesis, thereby boosting the seeding rate of anaerobic-sensitive varieties under flooded direct-seeded conditions [36]. After germination, rapid elongation of coleoptiles is important for conferring tolerance to submergence. Most recently, He et al. reported that the natural variation in rice coleoptile length subjected to submergence is determined by *UGT75A*, a glucosyl-transferase-encoding gene that decreases free ABA and jasmone acid (JA) levels by promoting the glycosylation of these two phytohormones under submergence conditions [37]. In addition, they found that UGT75A accelerates coleoptile elongation by inhibiting the interactions between JASMONATE ZIMDOMAIN (JAZ) and ABSCISIC ACID-INSENSITIVE (ABI) proteins. This study further highlights the negative effects of ABA signaling on AG/AGD in rice.

Rapid coleoptile elongation during submergence is mainly attributed to cell elongation. Auxin plays an essential role in this process [38]. MiR393 can shear the mRNA of *TRANSPORT INHIBI-TOR RESPONSE 1* (*TIR1*)/*AUXIN SIGNALING F-BOX 2* (*AFB2*) auxin receptors, which regulate *AUXIN RESPONSE FACTOR* (*ARF*) expression [39]. Submergence downregulates the expression of miR393, thus activating the auxin signaling pathway and stimulating coleoptile elongation in rice [40]. Interestingly, a recent report has shown that a certain threshold level of endogenous auxin is required for rice germination and early seedling growth during submergence [41]. Lee and his co-workers showed that a submergence-repressed miR167 is involved in the metabolic regulation of IAA through the miR167a-ARF-GH3 pathway [41]. MiR167-directed ARF mRNA cleavage is accompanied by the downregulation of *glycoside hydrolase 3* (*GH3*) [42]. *GH3-8* encodes an IAA-amido synthetase that prevents free IAA accumulation and maintains auxin homeostasis [43]. Reduced miR167 levels or increased GH3-8 levels reduce the endogenous levels of free IAA, thus promoting rice AG/AGD [41]. Recently, *OsPIN2*, a gene encoding an auxin efflux transporter, was found to negatively regulate hypoxia tolerance by affecting auxin transport and ROS accumulation [44]. These findings indicate that plant hormones are important regulators of AG/AGD in rice.

## 3. The Tolerance of Rice to Submergence at the Vegetative Stage and the Underlying Molecular Regulatory Mechanisms

### 3.1. Morphologic Adaptation to Submergence at the Vegetative Stage

In natural fields, from seedlings to mature plants for harvesting, rice is frequently subjected to submergence stress, which seriously compromises its growth, development, and yield. As a wetland plant species, rice possesses several adaptive traits, such as aerenchyma and adventitious roots, to cope with submergence stress.

#### 3.1.1. Aerenchyma Formation

The major adaptive feature of rice in response to submergence is the formation of aerenchyma, which is well-developed in the leaves, sheaths, roots, and internodes [2,10]. Ethylene triggers the formation of aerenchyma in maize and rice [5]. This aerenchyma constitutes gas spaces and becomes interconnected, which allows oxygen to transit from the above-water parts of the plant to the submerged roots. Generally, aerenchyma formation begins at the apical parts of the roots and gradually increases toward the basal parts of the roots [45]. There are two main types of aerenchyma: schizogenous and lysigenous. Lysigenous aerenchyma has been well studied, which can be constitutively formed in roots even under well-drained soil conditions, and its formation can be further induced under waterlogging conditions [46]. In addition, aerenchyma formation is less extensive in non-wetland plant species than in wetland plant species. Consistent with this, non-wetland plants are less tolerant to waterlogging than wetland plants such as rice [45,47]. Hence, the capacity to produce enough aerenchyma is important for increasing the tolerance to waterlogging in dryland crops [48,49].

#### 3.1.2. Adventitious Roots Formation

One of the most prominent outcomes of flooding is the low oxygen availability, which severely inhibits root growth. Limited oxygen availability impedes cell division and elongation, thus inhibiting the elongation of rice roots [50]. Under submerged conditions, rice root systems tend to become shallower and more compact, accompanied by an increase in adventitious roots (ARs) [10]. Adventitious roots reduce the distance between oxygen and the nutrient supply from the shoot. Pre-existing and newly formed ARs emerge upon submergence to replace the primary root system, which becomes easily dysfunctional when flooded under water. This adaptation enables plants to acquire oxygen and nutrients efficiently under flooded conditions. Recently, Lin et al. found that rice cultivar T65 produces only one type of AR, whereas NIL-12 (near-isogenic lines) enables the production of two types of ARs following partial submergence. Further morphological and anatomical traits suggest that AR2 (type 2 ARs, emergence several days later at the nodal region above type 1 ARs) is better adapted to flooding than AR1 (type 1 ARs, emergence from nodes early during submergence) [51].

#### 3.1.3. Shoot Elongation

Under flooded conditions, shoot elongation in rice is observed to be a response to flooding. Depending on the depth and duration of submergence, rice cultivars employ two different approaches to modulate shoot morphology. One of the approaches is the quiescence strategy, in which shoot elongation is arrested so that carbohydrates can be preserved for regrowth after floodwater recedes [4,6,52]. A comprehensive study performed on a group of 903 cultivars from the International Rice Research Institute provided strong evidence supporting the negative correlation between shoot elongation and submergence tolerance [53]. This deliberate reduction in shoot elongation allows these genotypes to effectively allocate resources and increase their probability of survival during the recovery stage [54]. Another approach is the escape strategy, which is characteristic of upland cultivars, in which the shoot/internode elongates rapidly to potentially grow above water [4,6,52]. Deepwater rice cultivars frequently use this approach to optimize survival under submerged conditions during the monsoon season [2].

### 3.2. Genetic Mechanism of Submergence Tolerance

**Figure 2:**
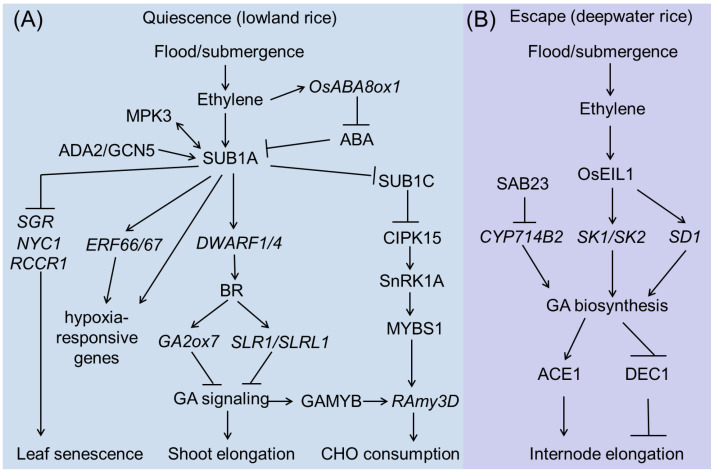
*SUB1A*-regulated (**A**) and *SKl/SK2*-regulated (**B**) submergence tolerance during rice vegetative stage.

The genetic basis of rice submergence tolerance at the vegetative stage is becoming increasingly clear. An overview picture is shown in Figure 2. ‘Quiescence’ and ‘Escape’ are two major and opposite strategies employed to counteract the effects of submergence by lowland rice and deepwater rice, respectively (Figure 2).

#### 3.2.1. *SUB1A* Pathway

Genetic mapping studies have associated the submergence-tolerant phenotype with a single QTL on chromosome 9 in FR13A, named *SUB1*. This QTL accounts for 69% of the phenotypic variation in rice flooding tolerance [55]. Further studies have shown that the *SUB1* locus contains three genes, *SUB1A*, *SUB1B*, and *SUB1C*, which encode proteins belonging to the Group VII ethylene-responsive factor (ERFVII) [17,19,56]. Most rice varieties contain *SUB1B* and *SUB1C* genes, whereas *SUB1A* only exists in flooding-tolerant varieties [15,57]. Upon submergence, *SUB1A* and *SUB1C* expression is rapidly induced; however, the expression of *SUB1B* is only slightly responsive [17]. Populations carrying *SUB1A* have two alleles, *SUB1A-1* and *SUB1A-2*. The only difference between the two alleles is the presence of a proline residue at position 186 in SUB1A-2, instead of a serine residue in SUB1A-1 [15,17,19,58]. *SUB1A-1* is specifically identified in submergence-tolerant cultivars, such as FR13A, and it can be strongly induced upon submergence to exert a determinant role in submergence stress responses, whereas the *SUB1A-2* allele, which is only found in submergence-sensitive cultivars, such as IR29, is poorly induced during submergence [15,58].

Under flooding conditions, ethylene inhibits internode growth in submergence-tolerant rice cultivars by inducing *SUB1A* expression [19]. As a transcription factor, SUB1A-1 can regulate a spectrum of genes involved in anaerobic metabolism, reactive oxygen species regulation, and leaf senescence during and after submergence by directly interacting with the GCC box in their promoters. During submergence, mitogen-activated protein kinase3 (MPK3) can specifically interact with and phosphorylate SUB1A-1 to activate it. Interestingly, activated SUB1A-1 can promote the expression of *MPK3* by binding to the GCC box in its promoter, thus forming a positive feedback loop during submergence [59]. SUB1A-1 also activates the transcription of other Group VII ERF transcription factors, such as *ERF66* and *ERF67*, and enhances submergence tolerance by regulating various hypoxia-responsive genes [60]. Recently, one report showed that SUB1A-1 can interact with ADA2b (ALTERATION/DEFICIENCY IN AC-TIVATION 2) of the ADA2b-GCN5 (GENERAL CONTROL NON-REPRESSIBLE 5) acetyltransferase complex and recruits this complex to modify the chromatin structure of the *ERF66/ERF67* promoter region and activate gene expression, which in turn enhances rice submergence tolerance [58]. In addition, the expression of *DWARF1* (*DWF1*) and *DWARF4* (*DWF4*), two key genes involved in the biosynthesis of brassinosteroids (BRs), is significantly higher in M202-Sub1 (tolerant variety) than in M202 (intolerant variety) during submergence, suggesting that SUB1A-1 can activate BR signaling. Consistent with the gene expression data, the SUB1A genotype has higher BR levels after submergence than those of the intolerant genotype. BRs in turn activate the transcription of the GA catabolic gene *GA2 oxidase7* (*GA2ox7*) or the protein accumulation of *slender rice-1* (*SLR1*) and *SLR1 like-1* (*SLRL1*), which negatively regulate GA responsiveness [61,62]. Apart from GA and BR signaling, the increase in ethylene levels also promotes the expression of *ABA 8′-hydroxylases* (*ABA8ox*), which stimulates the catabolism of bioactive ABA to unstable ABA. However, the presence or absence of SUB1A does not affect the decline in ABA levels in response to submergence, suggesting that growth inhibition in these submergence-tolerant plants is mainly brought about through the regulation of GA synthesis and signaling [63]. *SUB1A* overexpression also delays leaf yellowing and enhances recovery from dark stress. Physiological analysis revealed that SUB1A postpones dark-induced senescence by maintaining chlorophyll and carbohydrate reserves in the photosynthetic tissue. In line with this, RT-PCR results showed that SUB1A can downregulate the expression of senescence-associated genes (SAGs), including *stay-green* (*SGR*), *red chlorophyll catabolite reductase1* (*RCCR1*), and *non-yellow coloring1* (*NYC1*), when submerged [64].

Interestingly, the above shown SUB1A-mediated regulatory mechanisms are not usually active for anoxic seed germination [14], whereas the CIPK15 pathway is essential for this growth stage. But both SUB1A and CIPK15 play important roles in carbohydrate consumption in adult plants upon submergence. SUB1A can inhibit SUB1C, a positive regulator of the CIPK15-SnRK1A-MYBS1-RAmy3D pathway, resulting in limited growth under submerged conditions [65].

#### 3.2.2. *SK1/SK2* Pathway

In contrast to the quiescent strategy, deepwater rice adapts to submergence by rapidly elongating its internodes, thereby maintaining its leaves above the water surface. Through map-based cloning, another major submergence tolerance QTL of *qTIL12* in deepwater rice was cloned, which contains two ERF transcription factors genes: *SNORKEL1* (*SK1*) and *SNORKEL2* (*SK2*) [18]. *SK1/SK2* is not expressed under aerobic conditions; however, as early as 3 h of submergence treatment leads to their rapid accumulation. The expression of *SK1* and *SK2* is significantly induced by ethylene treatments, and overexpression of these genes promotes internode elongation in normal paddy rice, suggesting that SK1 and SK2 regulate internode elongation in response to ethylene [18]. SK1/SK2 cultivars can accumulate higher levels of GAs that promote internode elongation [18]. EIL1, a rice homolog of Arabidopsis EIN3, positively regulates ethylene response in rice [66]. A recent study showed that EIL1 can target the promoters of *SK1* and *SK2* to activate their expression.

*SEMIDWARF1* (*SD1*), encoding a GA biosynthesis enzyme, was identified as the *qTIL1* causative gene [67]. Two amino acid differences in SD1 exist between the deepwater rice variety C9285 and normal paddy rice, resulting in higher enzymatic activities in deepwater rice than in normal paddy rice. EIL1 can also directly bind to and activate *SD1* expression, thus promoting GA accumulation, particularly GA_4_, which promotes internode elongation. Recently, a gene in the SUB1A-binding protein family, *SAB23*, encoding a plant homeodomain (PHD)-type transcription factor, was identified as a novel negative regulator of submergence tolerance in rice. SAB23 binds to the promoter of *CYTOCHROME P450 714B2* (*CYP714B2*), which encodes a GA13-oxidase that catalyzes the conversion of GA_53_ to GA_19_, thus promoting the accumulation of bioactive GAs [68]. These findings clearly indicate that the activation of GA signaling is a key step in conferring submergence tolerance to deepwater rice.

Recently, an antagonistic regulatory mechanism of the GA-regulated stem growth in deepwater rice has been explored. *ACCELERATOR OF IN-TERNODE ELONGATION 1* (*ACE1*), which encodes a protein of unknown function, confers cells of the intercalary meristematic region with the competence for cell division, leading to internode elongation in the presence of GA. In contrast, *DECEL-ERATOR OF INTERNODE ELONGATION 1* (*DEC1*), which encodes a zinc finger transcription factor, suppresses internode elongation in response to GA [69]. More importantly, an analysis of genetic diversity suggests that mutations in *ACE1* and *DEC1* have historically contributed to the selection of shorter plants in domesticated populations of rice to increase their resistance to lodging, and of taller plants in wild species of rice to adapt to grow in deep water. Based on these findings, the regulation or modification of the GA pathway will be an efficient strategy for the molecular breeding of submergence-tolerant rice cultivars.

## 4. Conclusions and Perspectives

During evolution, rice has developed several different strategies to cope with flooding/submergence stress, which employ different genes or proteins to induce various morphological and physiological changes to survive and grow. During the seed germination and seedling establishment stages, CIPK15-mediated sugar signaling and the crosstalk between GA, ABA, JA, and auxin signaling commonly determine the tolerance of rice to submergence. At the vegetative growth stage, SUB1A functions as the central component of the submergence response that regulates multiple processes, such as entering a quiescent stage to preserve carbohydrate resources and helping the submerged plants acclimate during de-submergence. In contrast, SK1/SK2 promotes GA accumulation and triggers rapid shoot/internode elongation using carbohydrate reserves to escape flooding. Despite these exciting findings, the entire landscape of submergence tolerance in rice is not entirely depicted, and further exploration in the field is necessary to completely understand the mechanisms. In addition, the identification of genes that regulate submergence tolerance at both the seeding establishment and vegetative growth stages will be quite useful. In future, CRISPR-CAS9-mediated gene editing technology and molecular marker-assisted crossing breeding will be used to develop valuable commercialized varieties that harbor better tolerance to flooding.

## Data Availability

Not applicable.
